# Modern Diagnosis of Early Esophageal Cancer: From Blood Biomarkers to Advanced Endoscopy and Artificial Intelligence

**DOI:** 10.3390/cancers13133162

**Published:** 2021-06-24

**Authors:** Pierfrancesco Visaggi, Brigida Barberio, Matteo Ghisa, Mentore Ribolsi, Vincenzo Savarino, Matteo Fassan, Michele Valmasoni, Santino Marchi, Nicola de Bortoli, Edoardo Savarino

**Affiliations:** 1 Gastroenterology Unit, Department of Translational Research and New Technologies in Medicine and Surgery, University of Pisa, 56124 Pisa, Italy; p.visaggi@studenti.unipi.it (P.V.); s.marchi@med.unipi.it (S.M.); nicola.debortoli@unipi.it (N.d.B.); 2 Division of Gastroenterology, Department of Surgery, Oncology and Gastroenterology, University of Padua, 35121 Padua, Italy; brigida.barberio@unipd.it (B.B.); matteo.ghisa@aulss1.veneto.it (M.G.); 3 Department of Digestive Diseases, Campus Bio Medico University of Rome, 00128 Roma, Italy; m.ribolsi@unicampus.it; 4 Gastroenterology Unit, Department of Internal Medicine, University of Genoa, 16143 Genoa, Italy; vsavarin@unige.it; 5 Surgical Pathology & Cytopathology Unit, Department of Medicine (DIMED), University of Padua, 35121 Padua, Italy; matteo.fassan@unipd.it; 6 Department of Surgical, Oncological and Gastroenterological Sciences, Center for Esophageal Disease, University of Padova, 35124 Padova, Italy; michele.valmasoni@unipd.it

**Keywords:** diagnosis, esophageal cancer, early neoplasia, biomarkers, advanced endoscopy, artificial intelligence

## Abstract

**Simple Summary:**

Esophageal cancer (EC) has a poor prognosis when the diagnosis is delayed, but curative treatment is possible if the diagnosis is timely. The disease subtly progresses before symptoms prompt patients to seek medical attention. Effective pre-symptomatic screening strategies may improve the outcome of the disease. Recent evidence provided insights into early diagnosis of EC via blood tests, advanced endoscopic imaging, and artificial intelligence. Accordingly, we reviewed available strategies to diagnose early EC.

**Abstract:**

Esophageal cancer (EC) is the seventh most common cancer and the sixth cause of cancer death worldwide. Histologically, esophageal squamous cell carcinoma (ESCC) and esophageal adenocarcinoma (EAC) account for up to 90% and 20% of all ECs, respectively. Clinical symptoms such as dysphagia, odynophagia, and bolus impaction occur late in the natural history of the disease, and the diagnosis is often delayed. The prognosis of ESCC and EAC is poor in advanced stages, being survival rates less than 20% at five years. However, when the diagnosis is achieved early, curative treatment is possible, and survival exceeds 80%. For these reasons, mass screening strategies for EC are highly desirable, and several options are currently under investigation. Blood biomarkers offer an inexpensive, non-invasive screening strategy for cancers, and novel technologies have allowed the identification of candidate markers for EC. The esophagus is easily accessible via endoscopy, and endoscopic imaging represents the gold standard for cancer surveillance. However, lesion recognition during endoscopic procedures is hampered by interobserver variability. To fill this gap, artificial intelligence (AI) has recently been explored and provided encouraging results. In this review, we provide a summary of currently available options to achieve early diagnosis of EC, focusing on blood biomarkers, advanced endoscopy, and AI.

## 1. Introduction

In 2018 esophageal cancer (EC) was estimated to account for 508,000 deaths, being the seventh most common cancer and the sixth cause of cancer death worldwide [1]. Histologically, EC includes esophageal squamous cell carcinoma (ESCC) and adenocarcinoma (EAC). Usually, ESCC occurs in the middle or upper one-third of the esophagus, whereas EAC in the lower one-third or junction of the esophagus [2]. ESCC accounts for up to 90% of ECs in lower-income countries and in those regions spanning from Asian republics to north-central China, known as the “esophageal cancer belt” [3,4]. Complementarily, EAC accounts for around 20% of all ECs in Western Countries [3]. The replacement of esophageal squamous epithelium with intestinal metaplasia containing goblet cells defines Barrett’s esophagus (BE), which represents a well-known preneoplastic lesion for EAC. Interestingly, a recent meta-analysis revealed that around 12% of patients diagnosed with EAC had a prior BE diagnosis, and up to 57% of patients had concurrent diagnoses of BE and EAC [5].

Due to the late onset of clinical symptoms and the lack of early disease markers, EC is often diagnosed in advanced stages, when the prognosis is poor: ESCC has an overall 5-year survival rate of 18%, which decreases to less than 5%, when distant metastases are present at diagnosis [3]. Similarly, when EAC is diagnosed in advanced stages, the disease has a 5-year survival rate of less than 20% [1,6]. 

However, when early detection and management of EC is possible, the outcome improves significantly, and mortality decreases [7]. Therefore, several screening and preventive strategies are under investigation, each having its specific applicability, advantages, and disadvantages [8]. Of note, international guidelines do not currently include novel blood biomarkers, advanced endoscopy, or artificial intelligence (AI)-assisted endoscopy in the diagnostic work-up of EC. However, in recent years, the topic is increasingly being investigated, and a growing body of evidence is being provided. Accordingly, we reviewed the most recent literature addressing the early detection of ESCC and EAC via blood testing, advanced upper endoscopy, and novel AI systems. 

## 2. Literature Search

According to the aim of this narrative review, we provided an overview of the evidence from systematic reviews, meta-analyses, original research articles, reviews, and randomized controlled trials investigating the diagnosis of early EC. We conducted a literature review using the electronic databases PubMed, MEDLINE, EMBASE, and the Cochrane Library from inception to May 2021. The databases were searched combining the terms esophageal cancer AND diagnosis, blood biomarker, tumor marker, metabolite, protein, miRNA, lncRNA, circulating RNA, endoscopy, EGDS, advanced endoscopic imaging, artificial intelligence, computer-aided diagnosis. All terms were used as MeSH terms. Two authors independently reviewed all manuscripts retrieved from the literature research. The references of included studies were also reviewed to increase the source of information. All studies were included based on a consensus decision by the reviewing authors. 

## 3. Blood Biomarkers of Esophageal Cancer: A Liquid Biopsy

A biomarker is a biological molecule that can be found in the blood or in biological fluids or tissues of patients [9]. Blood biomarkers can be used for early diagnosis, prognosis, and clinical management of several cancer types [10]. There are some highly desirable characteristics that a cancer marker (CM) should possess, namely (i) high sensitivity in the screening of the general population, (ii) high specificity to a given type of tumor, (iii) be detectable in early cancers, providing a lead-time over clinical diagnosis, and (iv) correlate with the burden of a tumor, reflecting any tumor progression or regression [11]. Currently, the perfect CM does not exist, and circulating CMs recommended for clinical use are limited and include prostate-specific antigen, thyroglobulin, oncofetal antigens (e.g., carcinoembryonic antigen (CEA), alpha-fetoprotein) and carbohydrate antigens (CA) (e.g., CA125, CA19-9, CA15.3) [10]. 

Historically, CEA has been used as serum CM in the diagnosis of EC [12]. In this regard, CEA levels have been shown to be significantly higher in EC patients compared to controls [13]. In a meta-analysis [14], the sensitivity and specificity of CEA ranged from 8% to 70%, and from 57% to 100%, respectively, while its positive likelihood ratio (PLR) was 5.94 (95% confidence interval [CI], 3.24–10.89) meaning that patients with EC have a six-fold higher chance of having increased CEA levels compared to patients without EC [14]. The same study also investigated the diagnostic performance of squamous cell cancer antigen (SCC-Ag) and cytokeratin 21-1 fragment (CYFRA21-1) in the diagnosis of EC. The sensitivity and specificity Cyfra21–1 ranged from 36% to 63% and from 89% to 100%, respectively. The study revealed that patients with EC have a 12-fold higher chance of being Cyfra21–1 test-positive compared with patients without EC, having a PLR of 12.11 (95% CI, 5.02–29.24). As regards SCC-Ag, its sensitivity and specificity ranged from 13% to 64% and from 91% to 100%, respectively, whereas its PLR was 7.66 (95% CI: 4.24–13.83). 

More recently, the high level of technology of our era paved the way for novel substances that can be applied to the early detection of EC, providing insights into novel blood tests for screening and early diagnosis of EC (Table 1).

### 3.1. Blood Biomarkers of Esophageal Squamous Cell Carcinoma

Metabolomics uses gas chromatography-mass spectrometry (MS) to investigate the global fluctuation of molecular metabolites of <1500 Dalton in biofluids, cells and tissues [28]. In this regard, ESCC patients have been found to have a significant alteration of glucose, fatty acid metabolism, and tricarboxylic acid cycle compared to healthy controls [29]. Consistently, in 2018, Wang et al. [30] analyzed the metabolic profile of ESCC patients and identified that metabolite profiles in their serum were significantly different from those of healthy controls. In particular, a dysregulated lipid metabolism was found in ESCC patients, and phosphatidylcholines and choline kinase were identified as potential serum biomarkers of ESCC. In 2020, Zhang et al. [12] investigated serum metabolite changes in early-ESCC patients. The study demonstrated an activated synthesis of amino acids and inhibited desaturation of saturated fatty acids in ESCC patients compared to controls. Accordingly, a panel of four serum biomarkers including propanoic acid, linoleic acid, glycerol-3-phosphate, and L-glutamine showed an area under the curve (AUC) of 0.817, sensitivity and specificity of 75% and 74%, respectively, in the detection of esophageal squamous dysplasia (ESD). In the same study, the combination of serum levels of propanoic acid, L-leucine, and hydroxyproline showed AUC, sensitivity, and specificity of 0.817, 83%, and 74%, respectively, in the discrimination between ESCC and ESD. Similarly, Yang et al. [23] identified six serum metabolites as potential biomarkers of early ESCC, namely α-glucose, choline, glutamine, glutamate, valine, and dihydrothymine. The AUC of this panel in the diagnosis of early ESCC was 0.969, whereas the combination of serum levels of α-glucose, pyruvate, glutamate, and valine could distinguish ESCC from post-operative ESCC patients with an AUC of 0.985. 

Fan et al. [31] applied proteomics studies and MS for the protein profiling of sera of ESCC patients. The authors evaluated the differences between molecular mass peaks in the serum proteome profiles of ESCC patients and healthy subjects, identifying a five peptide peptidome that could diagnose ESCC with 96.7% sensitivity, 100% specificity, and 98.4% accuracy. 

Tumor-derived microRNAs (miRNAs) are resistant to endogenous ribonuclease activity and are found in human serum in a stable form [32]. Therefore, such small molecules have been investigated as serum markers of EC [15,16,17]. A recent meta-analysis, including 35 studies investigating plasma or serum miRNAs of ESCC patients [33], concluded that circulating miRNAs could distinguish ESCC patients from controls with a pooled sensitivity of 79.4% (95% CI, 76.5–82.0%), specificity of 77.9% (95% CI, 74.6–80.8%), and AUC of 0.86 (95% CI, 0.82–0.88). Another meta-analysis exclusively focusing on Asian populations found that miRNAs could distinguish patients with ESCC from controls with 77.7% pooled sensitivity (95% CI, 74.2–80.9%), 80.9% specificity (95% CI, 76.6–84.6%), and AUC of 0.86 (95% CI, 0.83–0.89) [34].

Long non-coding RNAs (lncRNAs) have also been investigated as potential biomarkers for ESCC. LncRNAs are circulating genetic material that has no coding ability but can regulate gene expression and play a role in tumorigenesis [35]. Among investigated lncRNAs, POU3F3 demonstrated an AUC of 0.842, a sensitivity of 72.8%, and a specificity of 89.4% in the diagnosis of ESCC [22]. In the same study, the combination of POU3F3 and SCCA achieved an AUC of 0.926, a sensitivity of 85.7%, and a specificity of 81.4% in the detection of early-stage ESCC [22]. In another study on lncRNAs [21], the combination of Linc00152, CFLAR-AS1, and POU3F3 with CEA achieved an AUC of 0.955. 

### 3.2. Blood Biomarkers of Esophageal Adenocarcinoma

Similar to ESCC, no tissue or circulating CM has been approved for clinical use in the diagnosis of EAC. However, recent studies have shed light on possible novel blood screening tests. Fassan et al. [18] have recently proposed circulating miRNAs as biomarkers in the follow-up of BE patients. In particular, a significant upregulation of 10 miRNAs (miR-92a-3p, miR-151a-5p, miR-362-3p, miR-345-3p, miR-619-3p, miR-1260b, and miR-1276), and downregulation of three miRNAs (miR-381-3p, miR-502-3p, and miR-3615) was found in patients with early EAC compared to patients with non-dysplastic BE (NDBE). Accordingly, Chiam et al. [19] developed a panel of several miRNAs ratios that showed an AUC of 0.99 (95% CI, 0.96–1.0) for the distinction between EAC from NDBE (RNU6-1/miR-16-5p, miR-25-3p/miR-320a, let-7e-5p/miR-15b-5p, miR-30a-5p/miR-324-5p, miR-17-5p/miR-194-5p). Zhang et al. [20] profiled the circulating miRNAs of patients with EAC and found significant differences compared to healthy controls. In contrast, Craig et al. [36] failed to identify a serum miRNA signature of EAC. 

As immune imbalance and inflammation seem to play a role in the progression from BE to EAC, Campos et al. [26] investigated the neutrophil-lymphocyte ratio (NLR) in BE and EAC patients. The authors found that NLR progressively increased and significantly correlated with the presence of dysplasia or neoplasia (*p* < 0.001). Additionally, a NLR > 2.27 detected EAC with 80% sensitivity, 71% specificity, and 0.8 AUC. In another study, a panel of 10 serum glycoproteins distinguished BE from EAC with an AUC of 0.93 [37]. 

Through the phosphatidylinositol glycan class-A (PIG-A) gene mutation assay, Haboubi et al. [27] evaluated the erythrocyte mutant frequency (EMF) as a marker of genomic instability in EAC. The authors collected blood samples of patients undergoing upper endoscopy and fluorescently stained erythrocytes for glycosylphosphatidylinositol (GPI)-anchored proteins. GPI-anchor negative erythrocytes were considered mutants, and the EMF was calculated through flow cytometry. EAC patients showed a three-fold increase in EMF compared to controls (*p * <  0.001), thus suggesting that the PIG-A gene mutation assay could be applied to early detection of EAC.

### 3.3. Serum Autoantibodies in Esophageal Squamous Cell Carcinoma and Adenocarcinoma 

The study of the immune system may be of help in cancer screening because there is evidence of an immune response to cancer in humans due to the presence of autoantibodies against intracellular and surface antigens in cancer patients [38,39,40]. In this regard, antibodies directed against circulating tumor-associated antigens have been demonstrated to be present in the serum of patients many years before the diagnosis of cancer and might be a useful non-invasive tool in cancer screening [24,25]. Accordingly, serum antibodies against p53 demonstrated a statistically significant relationship with the subsequent development of malignancy with an average lead time to diagnosis of 3.5 years in lung cancer [41,42] and have been recently associated with tumor progression in ESCC [43]. In 2015, a systematic review [4] investigated the role of autoantibodies in the diagnosis of ESCC and EAC. Anti-p53, anti-p16, anti-cyclin B1, anti-c-Myc, anti-HSP70, and anti-LY6K were found to be the most relevant. The sensitivity of single antibodies ranged from 3.9% to 93.7% and the specificity from 78.7–100%. The highest sensitivities were found for antibodies against HSP70 (93.7%) and the anti-HSP70 antibodies had the highest specificity of 100%. Similarly, the sensitivity of anti-LY6K antibodies was more than 80%, but it had the lowest specificity of 78.7%. As to the combinations of autoantibodies in the diagnosis of EC, their sensitivity ranged from 22.5% to 86% (median 54.3%), whereas the specificity ranged from 89% to 100% (median 95.1%) [4]. 

In summary, several emerging technologies have been applied to the blood screening of EC achieving good performance in the detection of both ESCC and EAC. However, most studies involved small samples of patients and did not include multiple research centers. Mass screening via blood sampling would be an ideal strategy to reduce the high costs and low effectiveness of endoscopy screening for EC, but further multi-center, prospective, and large-scale studies are needed to identify high-performing blood markers for EC to be used in routine practice. 

## 4. Advanced Endoscopic Imaging in the Diagnosis of Esophageal Cancer

Esophagogastroduodenoscopy (EGDS) is the gold standard test for EC and its precursor lesions [44]. At present, dysplasia and cancer surveillance in BE follows the Seattle protocol with random 4-quadrant biopsies every 2 cm, which is expensive, time-consuming, and has a sensitivity ranging from 28% to 85% for the detection of high-grade dysplasia (HGD)/EAC [45]. These drawbacks contribute to <50% adherence to the Seattle protocol in clinical practice [46]. Unlike BE, random sampling in ESCC screening would be unpractical because the entire esophageal mucosa can harbor ESD, and this requires extensive biopsy sampling [44]. Moreover, endoscopic recognition of early ESCC is challenging, as lesions often pass unrecognized with standard WLE, which may miss up to 40% of early ESCC even in high-risk populations [47]. Accordingly, the sensitivity of targeted biopsies for ESD may be as low as 7.7% [48]. 

In order to improve the diagnostic yield of standard WLE procedures, novel endoscopic techniques have been investigated, namely dye spray chromoendoscopy, virtual chromoendoscopy (VCE), confocal laser endomicroscopy (CLE), and volumetric laser endomicroscopy (VLE) (Table 2).

### 4.1. Dye Spray Chromoendoscopy 

Chromoendoscopy is a technique that involves the spraying of dyes on the luminal esophageal surface to obtain selective mucosal uptake (vital staining, i.e., methylene blue, Lugol’s iodine solution) or mucosal pattern enhancement (contrast staining, i.e., indigo carmine, acetic acid (AA)) [53]. 

The topical application of 1.5–2.5% AA stains NDBE white, while early EAC loses the aceto-whitening within a few seconds [44]. A recent meta-analysis including nine studies and 1379 patients [49] found that AA chromoendoscopy had a pooled sensitivity of 92% (95% CI, 0.83–0.97), pooled specificity of 96% (95% CI, 0.85–0.99), positive likelihood ratio (LR) of 25.0 (95% CI, 5.9–105.3), and negative LR of 0.08 (95% CI, 0.04–0.18) for the diagnosis of HGD and EAC. The diagnostic performance of AA chromoendoscopy may be up to six-fold higher than the Seattle protocol (*p* = 0.0001) [54]. Conversely, a meta-analysis of eight studies concluded that there was no incremental yield with methylene blue chromoendoscopy over standard four-quadrant biopsies for the detection of HGD or EAC [55].

Lugol’s iodine dye spray is used in the diagnosis of ESCC as squamous neoplasia appears as Lugol-voiding lesion (LVL) [44]. Additionally, discoloration of the LVL within 3 min from the staining is referred to as pink sign and significantly correlates with HGD and EAC on histology [56]. A recent meta-analysis assessed the diagnostic performance of Lugol chromoendoscopy (LC) in 12 studies and 1911 patients [50]. In per-patient analysis, LC showed a sensitivity of 92% (95% CI, 86–96%), specificity of 82% (95% CI, 80–85%), positive LR of 5.42 (95% CI, 3.21–9.13), and negative LR of 0.13 (95% CI, 0.08–0.23).

However, the use of chromoendoscopy is associated with drawbacks in clinical practice, including the need for dedicated equipment, impossibility to study superficial vascularity, difficulty to obtain uniform mucosal coating, and the duration of the procedure [53]. Moreover, dye spraying carries the risk of allergic reaction to the dye, aspiration and pneumonia, and chest discomfort [50]. For these reasons, dye spray chromoendoscopy is being increasingly replaced by virtual chromoendoscopy (VCE).

### 4.2. Virtual Chromoendoscopy 

The term VCE refers to endoscopic imaging techniques that provide contrast enhancement of the mucosal surface and blood vessels without the use of stains or dyes [57]. This technology is based on the observation that visualization of some mucosal tissues is wave-length dependent. Selective light transmittance is accomplished by the optical filtering of white light in narrow-band imaging (NBI), and with post-image processing in flexible spectral imaging color enhancement (FICE) and i-SCAN [57]. Recently, blue laser imaging (BLI), which utilizes two monochromatic lasers at 410 nm and 450 nm, has become available [44]. However, currently available studies on FICE, i-SCAN, and BLI in the diagnosis of EC are limited and appeared to be of controversial utility [58,59,60]. Therefore, further studies are needed to assess whether such techniques could be incorporated in clinical practice for EC diagnosis. 

Conversely, NBI significantly increases the specificity of endoscopic procedures compared to Lugol’s dye in the diagnosis of ESCC (per-patient and per-lesion specificities: 88% and 65% versus 82% and 37%, respectively), with comparable sensitivity (per-patient and per-lesion sensitivities: 88% and 94% versus 92% and 98%, respectively) [50]. Moreover, a meta-analysis assessing the performance of NBI in the diagnosis of dysplasia in BE [51] concluded that the use of NBI during EGDS showed a pooled sensitivity of 94.2% (95% CI, 82.6–98.2), specificity of 94.4% (95% CI, 80.5–98.6), and negative predictive value of 97.5% (95% CI, 95.1–98.7). Additionally, a meta-analysis on advanced imaging techniques for the identification of dysplasia or cancer in patients with BE showed that VCE significantly increased the diagnostic yield compared to standard endoscopy [61].

### 4.3. Confocal Laser Endomicroscopy

Probe-based CLE (pCLE) provides both surface and subsurface images of esophageal mucosa, capturing near-microscopic images at approximately 150 µm of depth via a flexible mini-probe introduced through the accessory channel of standard endoscopes [62]. However, the endoscope-integrated system for CLE (eCLE), which provided images at different depths from 0 to 250 mlm and allowed a free accessory channel for esophageal biopsy, is no longer available on the market [62]. CLE investigations require contrast agents, including intravenous fluorescein sodium or topical acriflavine hydrochloride and cresyl violet. When fluorescein is administered intravenously, it stains the extracellular matrix of the surface epithelium, whereas topical acriflavine and cresyl violet stain the nuclei of superficial layers of the mucosa [62]. In 2011, the Miami consensus classification of CLE provided guidelines to distinguish normal squamous epithelium, BE with and without dysplasia and intramucosal carcinoma [63]. Subsequently, CLE has been mainly applied to the detection of BE-related dysplasia. A systematic review with meta-analysis by the American Society for Gastrointestinal Endoscopy (ASGE) Technology Committee concluded that, in expert hands, the pooled sensitivity, NPV and specificity of CLE in the detection of BE dysplasia were 90.4% (95% CI, 71.9–97.2), 98.3% (95% CI, 94.2–99.5), and 92.7% (95% CI, 87–96), respectively [51]. Conversely, the lack of consensus on the use of pCLE in ESCC limits the application of the technique in clinical practice [44]. However, one study evaluating pCLE in the diagnosis of ESCC showed up to 95% accuracy, 100% sensitivity, and 87% specificity [64].

Although CLE has proven utility in the diagnosis of EC, its routine use appears hardly applicable to daily clinical practice because of its time-consuming nature, high costs, and high level of expertise needed for proper interpretation. 

### 4.4. Volumetric Laser Endomicroscopy (Optical Coherence Tomography) 

VLE is a new advanced endoscopic imaging technology based on optical coherence tomography (OCT). A balloon-based system generates a circumferential scan of 6 cm segments of the esophagus to a depth of 3 mm into the mucosal and submucosal layers with 7 µm axial resolution [65]. Unlike CLE, VLE does not require intravenous contrast agents [62]. In 2017, VLE diagnostic criteria for the diagnosis of squamous esophagus, NDBE and BE was proposed [66]. Historically, VLE has been mainly applied to BE. However, due to the long esophageal segments included in the field of view of VLE, the technique may be suitable for screening and surveillance of ESCC. A systematic review assessing the performance of OCT in the diagnosis of EC [52] concluded that VLE had a sensitivity ranging from 81% to 97% and specificity from 57% to 92%. In the detection of dysplasia and early EC, the sensitivity was 68–83% and specificity 75–82%. In the staging of early ESCC, OCT showed an accuracy of >90%. Additionally, a single study compared the performance of VLE and pCLE in the detection of esophageal dysplasia [67]. The diagnostic accuracy of VLE was 87%, which was significantly superior to that of pCLE. Based on available evidence, OCT has the potential to support screening and diagnosis of EC. However, further prospective and large sample-size studies are needed to confirm these early results. 

## 5. Artificial Intelligence in the Diagnosis of Esophageal Cancer

The ability to recognize endoscopic images depends on individual expertise, being inter- and intra-observer variability that limits endoscopic procedures. AI is being extensively applied to upper GI diseases aiming to improve the diagnostic yield of endoscopy. The term AI generically refers to complex computer algorithms that mimic human cognitive functions, including learning and problem solving [68]. Machine learning (ML) and deep learning (DL) represent a form of AI in which computer algorithms are used to recognize discriminative features of data and provide appropriate outputs (i.e., elaborating information to provide a diagnosis) [68]. Numerous systems of computer-aided diagnosis (CAD) have been recently tested in upper GI endoscopy showing encouraging results. AI has the potential to successfully assist both trainees and expert physicians to reduce variability in the detection of esophageal cancer, thus increasing the diagnostic accuracy regardless of individual expertise and virtually overcoming the current limitations of EGDS [69]. 

Advanced endoscopic imaging techniques have shown an accuracy in the detection of EC. As to ESCC, VCE with NBI and blue-laser imaging (BLI) improves the recognition of lesions [70,71]. As regards EAC, the American Society for Gastrointestinal Endoscopy (ASGE) recently endorsed targeted biopsies with AA chromoendoscopy, NBI, and eCLE during surveillance of patients with previous NDBE because these techniques can meet the optical diagnosis performance required by the Preservation and Incorporation of Valuable Endoscopic Innovations (PIVI) initiative by ASGE (i.e., per-patient sensitivity of 90% or greater, a negative predictive value (NPV) of 98% or greater, and a specificity of at least 80% for detecting HGD or EAC) [45,51]. 

Notably, advanced endoscopic imaging techniques require much training [71], which limits the extensive use in clinical practice. In this regard, the support of AI tools is being investigated and has already provided promising results in meta-analytic studies (Table 3). 

In the diagnosis of ESCC, the pooled sensitivity, specificity, positive predictive value (PPV), and negative predictive value (NPV) of AI with WLE and/or NBI were 93% (CI 73% to 99%), 89% (CI 77% to 95%), 77% (CI 55% to 89%), and 97% (CI 88% to 100%), respectively [72]. In the diagnosis of EAC, on the other hand, the pooled sensitivity, specificity, PPV, and NPV of AI with WLE and/or NBI were 89% (CI 83% to 93%), 88% (CI 84% to 91%), 88% (CI 84% to 91%), and 89% (CI 83% to 93%), respectively. 

In another meta-analysis [74], the overall pooled performance of AI in the detection of ESCC with NBI, WLE, endo-cytoscopy and ME was: AUC 0.88 (95% CI, 0.82–0.96), sensitivity 75.6% (95% CI, 48.3–92.5%), and specificity 92.5% (95% CI, 66.8–99.5%). In the diagnosis of ESCC, AI performed significantly better with NBI compared to WLE, achieving an AUC of 0.92 (95% CI, 0.86–1.00) and 0.83 (95% CI, 0.82–0.84), respectively. For the detection of EAC, it was estimated that AI could make a diagnosis with pooled AUC, sensitivity, and specificity of 0.96 (95% CI, 0.93–0.99), 88% (95% CI, 82.0–92.1%), and 90.4% (95% CI, 85.6–94.5%), respectively, when applied to WLE and VLE. Notably, AI performed significantly better than endoscopists in the diagnosis of EAC, demonstrating AUC, sensitivity, and specificity of 0.96 (95% CI, 0.94–9.97) vs. 0.82, 90.7% (95% CI, 89.8–91.5%) vs. 72.3% (95% CI, 70.2–74.3%), and 88.0% (95% CI, 87.1–88.9%) vs. 74.0% (95% CI, 72.2–75.7%), respectively.

Two meta-analyses evaluated the overall performance of AI in the diagnosis of esophageal cancer regardless of histology and endoscopic light [73,75]. For the detection of EC, Bang et al. estimated a pooled AUC, sensitivity, and specificity of 0.97 (95% CI, 0.89–0.96), 94% (95% CI, 89–96%), and 88% (95% CI, 76–94%), respectively, whereas Mohan et al. estimated a pooled accuracy, sensitivity, specificity, PPV, and NPV of 87.2% (95% CI 76–93.6), 87.1% (95% CI 69.4–95.3), 87.3% (95% CI 74.3–94.2), 72.3% (95% CI 41.7–90.5), and 92.1% (95% CI 85.9–95.7), respectively. 

Although the performance of AI was proven to be high in several meta-analyses, the majority of included studies were retrospective and used still endoscopic images to test the AI system. More recent single studies investigated the performance of AI during real-time endoscopic procedures. 

In the diagnosis of ESCC from NBI video clips, AI achieved accuracy, sensitivity, and specificity of 63%, 51%, and 91%, respectively [76]. In the same task, thirteen expert endoscopists achieved accuracy, sensitivity, and specificity of 75%, 72% and 79%. In the differentiation of cancerous from noncancerous lesions, AI had significantly better diagnostic performance than endoscopists, with a sensitivity of 86% vs. 74%, specificity of 89% vs. 76%, and accuracy of 88% vs. 75%. 

In the diagnosis of EAC and NDBE from NBI zoom video clips, AI showed accuracy, sensitivity, and specificity of 83%, 85%, and 83%, respectively [77]. In another study, de Groof et al. assessed the accuracy of a CAD system for the detection of Barrett’s neoplasia within endoscopic images systematically taken every 2 cm in Barrett’s areas during live endoscopic procedures [78]. 

During the EGDS, the AI system in real-time achieved 90% accuracy, 91% sensitivity, and 89% specificity, thus meeting PIVI thresholds. Finally, in another study [79], AI distinguished EAC from NDBE with 89.9% accuracy, 83.7% sensitivity, and 100% specificity from endoscopic images that were randomly captured from the camera live-stream during endoscopy. 

Recent studies tested AI in the characterization of EC. As regards ESCC, AI has been applied to the detection of esophageal intrapapillary capillary loops (IPCLs) and invasion depth estimation. IPCL are micro-vessels on the surface of the esophagus that appear as brown loops on magnifying endoscopy (ME) with NBI, whose morphological changes correlate with the invasion depth of ESCC, allowing intra-procedural decisions for endoscopic resections [80,81]. Everson et al. [82] developed a DL system that detected abnormal IPCL with 93.7% accuracy, 89.3% sensitivity, and 98% specificity. Herrera et al. [83] developed a DL algorithm that detected abnormal IPCLs with 91.7% accuracy, 93.7% sensitivity, and 92.4% specificity, respectively, thus performing as well as experts in the field. The AI algorithm of Tokai et al. [84] estimated the invasion depth of ESCC images under NBI and WLE with 80.9% accuracy, 84.1% sensitivity, and 73.3% specificity, thus performing significantly better than expert endoscopists who achieved accuracy, sensitivity, and specificity of 73.5%, 78.8%, and 61.7%, respectively. Nakagawa et al. [85] developed an AI system that differentiated mucosal and submucosal microinvasive (SM1) from the submucosal deep invasive (SM2/3) cancers with 91.0% accuracy, 90.1% sensitivity, and 95.8% specificity. 

As regards the characterization of lesions in EAC, in a pilot study, AI could predict submucosal invasion and differentiated stage T1a from T1b in endoscopic images of Barrett’s cancer with 71.0% accuracy, 77.0% sensitivity, and 64.0% specificity. Importantly, the performance of AI was comparable to that of international experts in the field [86]. 

## 6. Conclusions

EC is a highly aggressive malignancy due to late diagnosis, rapid progression, and poor prognosis of survival. However, curative treatment is possible when the disease is diagnosed early. 

The incidence of EAC is increasing in Western countries, likely due to widespread risk factors as gastro-esophageal reflux, central obesity, and increasing age [62]. Although the incidence of ESCC has declined in China in recent years, the absolute incidence is high because of the large population [35]. Accordingly, screening strategies for a timely diagnosis are needed to reduce the mortality of EC patients. Current guidelines do not address the use of novel biochemical markers, recently introduced advanced endoscopic imaging techniques or AI tools as screening for EC [87,88,89,90,91]. This is largely due to the lack of high-quality evidence, the heterogeneity of available studies, and to the fact that highly sophisticated technologies have been developed only recently. In this regard, a growing body of evidence on the use of blood tests, advanced and AI-assisted endoscopy is now becoming available, offering new insights into what the diagnosis of EC may look like in the near future. Accordingly, we reviewed the most recent evidence that could possibly contribute to innovate the diagnosis of EC.

Several blood-based biomarkers have been investigated. Circulating molecules that have shown potential utility include CEA, Cyfra21-1, SCC-Ag, metabolites, proteins, NLR, autoantibodies, and circulating RNAs (circRNAs). In this regard, a recent meta-analysis [92] concluded that circRNAs have a pooled sensitivity of 79% (95% CI: 0.69–0.87), specificity of 85% (95% CI: 0.68–0.94), overall positive likelihood ratio of 5.27 (95% CI: 2.46–11.32), negative likelihood ratio 0.24 (95% CI: 0.16–0.36), diagnostic odds ratio 21.66 (95% CI: 9.33–50.30), and AUC of 0.88 (95% CI: 0.84–0.90) in the diagnosis of EC. In summary, liquid biopsies appear as promising non-invasive inexpensive tests for the diagnosis of EC, and further investigations are required to extensively disclose their clinical utility. 

EGDS represents the test of choice for investigations on EC [44]. Several advanced endoscopic imaging techniques are currently available to improve the recognition of lesions during procedures, namely dye spray chromoendoscopy, VCE, CLE, and VLE. Meta-analytic studies confirmed the superiority of advanced endoscopy compared to standard WLE in the diagnosis of EC [50,61]. However, their routine application to clinical practice is hampered by the high level of expertise required for their use. 

In recent years, CAD tools are providing an interpretable universal method for endoscopic diagnosis, virtually eliminating variability among observers. Recent meta-analyses [72,73,74,75] confirmed the potential of AI to increase the diagnostic yield and reduce underdiagnosis of upper GI neoplastic lesions, often performing comparably or better than expert endoscopists. However, most studies on CAD tools were retrospective and tested the diagnostic yield of AI on endoscopic images rather than during live EGDS. Accordingly, available evidence should be interpreted with caution. More real-time, high-quality studies are needed to confirm and expand these early results and allow the integration of AI into medical workflows to anticipate the diagnosis of EC.

## Figures and Tables

**Table 1:** Potential circulating blood molecules in the screening of esophageal cancer.

Type of Biomarker	Disease	Panel
miRNA	ESCC [15,16,17]	miR-25, miR-100, miR-193-3p, miR-194, miR-223, miR-337-5p, miR-483-5p
		miR-10a, miR-22, miR-100, miR-148b, miR-223, miR-133a, miR-127-3p
		MiR-21, miR-375
	EAC [18,19,20]	miR-92a-3p, miR-151a-5p, miR-362-3p, miR-345-3p, miR-619-3p, miR-1260b, and miR-1276
		RNU6-1/miR-16-5p, miR-25-3p/miR-320a, let-7e-5p/miR-15b-5p, miR-30a-5p/miR-324-5p, miR-17-5p/miR-194-5p
		miR-25-3p, miR-151a-3p, miR-100-5p, miR-375
lncRNA	ESCC [21,22]	POU3F3, SCCA
		Linc00152, CFLAR-AS1, POU3F3
Metabolite	ESCC [12,23]	propanoic acid, linoleic acid, glycerol-3-phosphate, and L-glutamine
		propanoic acid, L-leucine, and hydroxyproline
		α-glucose, choline, glutamine, glutamate, valine, and dihydrothymine
Antibody	ESCC [24]	Antibody against p53, NY-ESO-1, MMP-7, Hsp70, Prx VI, Bmi-1
	EAC [25]	Antibody against amino acid L-proline, ketone body 3-hydroxybutyrate, carbohydrate D-mannose
	ESCC/EAC [4]	anti-p53, anti-HSP70, anti-p16, anti-cyclin B1, anti c-Myc, anti-LY6K
Blood cells	EAC [26,27]	Neutrophil-lymphocyte ratio
		Erythrocyte mutant frequency

Abbreviations: miRNA, micro-RNA; lncRNA, long non-coding RNA; ESCC, esophageal squamous cell carcinoma; EAC, esophageal adenocarcinoma.

**Table 2:** Performance of advanced endoscopic imaging in the diagnosis of esophageal cancer according to systematic reviews and meta-analyses.

Author	Endoscopic Technique	Disease	Sensitivity (95% CI, %)	Specificity (95% CI, %)
Coletta et al. [49]	AA Chromoendoscopy	EAC	92% (83 to 97)	96% (85 to 99)
Morita et al. [50]	Lugol Chromoendoscopy	ESCC	92% (86 to 96)	82% (80 to 85)
Thosani et al. [51]	NBI	EAC	94.2% (82.6 to 98.2)	94.4% (80.5 to 98.6)
Morita et al. [50]		ESCC	88% (86 to 93)	88% (86 to 90)
Thosani et al. [51]	pCLE *	EAC	90.4% (71.9 to 97.2)	92.7% (87 to 96)
Kohli et al. [52]	VLE ^#^	EC	81–97%	57–92%

* Performance refers to detection of dysplasia; ^#^ evidence from a systematic review without meta-analysis. Abbreviations: AA, acetic acid; NBI, narrow-band imaging; pCLE, probe-based confocal laser endomicroscopy; VLE, volumetric laser endomicroscopy; ESCC, esophageal squamous cell carcinoma; EAC, esophageal adenocarcinoma; EC, esophageal cancer.

**Table 3:** Performance of artificial intelligence in the diagnosis of esophageal cancer according to meta-analyses.

Author	Disease	Endoscopic Light	AUC (95% CI)/Accuracy (95% CI, %)	Sensitivity (95% CI, %)	Specificity (95% CI, %)	PPV (95% CI, %)	NPV (95% CI, %)
Arribas et al. [72]	ESCC	WLE and/or NBI	-	93% (73 to 99)	89% (77 to 95)	77% (55 to 89)	97% (88 to 100)
	EAC		-	89% (83 to 93)	88% (84 to 91)	88% (84 to 91)	89% (83 to 93)
Bang et al. [73]	ESCC or EAC	WLE and/or NBI	0.97 (0.89–0.96)	94% (89 to 96)	88% (76 to 94)	-	-
Lui et al. [74]	ESCC	WLE and/or NBI or ECS or ME	0.88 (0.82–0.96)	75.6% (48.3 to 92.5)	92.5% (66.8 to 99.5)	-	-
	EAC	WLE or VLE	0.96 (0.93 to 0.99)	88% (82.0 to 92.1)	90.4% (85.6 to 94.5)	-	-
Mohan et al. [75]	ESCC or EAC	WLE and/or NBI	87.2% (76–93.6)	87.1% (69.4 to 95.3)	87.3% (74.3 to 94.2)	72.3% (41.7 to 90.5)	92.1% (85.9 to 95.7)

Abbreviations: AUC, area under the curve; CI, confidence interval; EAC, esophageal adenocarcinoma; ECS, endocytoscopic system; ESCC, esophageal squamous cell carcinoma; ME, magnifying endoscopy; NBI, narrow band imaging; NPV, negative predictive value; PPV, positive predictive value; VLE, volumetric laser endomicroscopy; WLE, white light endoscopy.
